# Medical Jurisprudence of Insanity

**Published:** 1854-07-01

**Authors:** 


### MEDICAL JURISPRUDENCE OF INSANITY.

The common law (which is ours, except so far as we have modified it by the statutes) has adopted two widely different rules on the subject of insanity; one having relation to civil affairs, and the other referring entirely to criminal cases. By the first, a man whose mind is deranged, his intellects having become insufficient to conduct the common business of life, his property will be taken from him, and trustees appointed to take care and manage his estate. By the second, strange as it may seem, the same man, who has been adjudged incapable of conducting his own concerns on account of insanity, may be held responsible for criminal acts, provided he possesses a mind capable of distinguishing right from wrong. In legal effect there are, therefore, two kinds of unsoundness of mind—an unsoundness which is partial, and destroys one's capacity for civil affairs, and an unsoundness which is total, and utterly destroys the moral responsibility, so that the deranged is no longer a reasonable and accountable being. In contemplation of law, partial insanity simply reduces a man to the condition of a child, a minor under age, who cannot be compelled to fulfil his contracts, but who is still answerable for crimes committed. His position is similar to that of the habitual drunkard—he is deprived of the management of his property, because manifestly disqualified by his habits to take care of it judiciously; and similar, also, to that of the man whose mind falls into decay by reason of advanced age, and the apparent failure of the mental power.

According to the early writers, to excuse a man from the consequences of his act, he must have been, at the period when he committed the offence, wholly incapable of distinguishing between good and evil, or comprehending the nature of what he was doing. If he be but partially insane, the law does not excuse him, but holds him to a rigid accountability: making it necessary for him to show that, at the time the deed was committed, he was absolutely incapable of distinguishing between right and wrong. As Lord Hale, one of the sages of the law, expresses it, if he possess as great understanding as ordinarily a child of fourteen years hath, he may be guilty of treason or felony. It is well known to most of our readers, that the principles of what is termed the common law are ascertained from the decisions of the courts: our own first, and those of England secondly, by way of illustration. The reason of this is found in the fact that we hold our laws, like our literature and language, in common with that country, having derived them thence with our very being. For as the statesmen of the revolution contended, the men who first emigrated to this country brought with them the rights of freemen, and the laws and privileges of their own country. Instead of coming forth a loose, disjointed and confused congregation of reckless men, like the Spanish into Mexico and Peru, impatient of control, and thirsting for gold, they came forth freely and soberly, a well-appointed community. In place of an arbitrary government of undefined civil and military powers, they brought with them charters of liberty, civil officers, an organized government, and a society firmly knit together, wearing as a garment the common law of England. When, therefore, we quote the decisions of the English courts, they are not referred to as binding precedents, and authority to which we must yield obedience, but rather as the historical evidence of what the law was, or still continues to be. They are, as Coke termed, the witnesses of the law, to whose testimony on the subject of insanity we will now briefly refer.

In the case of Edward Arnold, indicted and tried at the Surrey Assizes, in England, for shooting at Lord Onslow, in 1724, the court, in charging the jury, used these words:—"It is not every kind of frantic humour, or something unaccountable in a man's actions, that points him out to be such a madman as is to be exempted from punishment; it must be a man that is totally deprived of his understanding and memory, and doth not know what he is doing, no more than an infant, than a brute, or a wild beast: such an one is never the object of punishment." Upon this charge it is scarcely necessary to say the jury found the prisoner guilty, and he received the sentence of death, though there was no question of his partial insanity. It is worthy of remark, that at the period of this trial, the accused, in such cases, were not allowed to come into court with counsel, except upon the special grace and favour of the court. In the case of Earl Ferrers, tried and convicted of the murder of John Johnson, in 1760, the same rule was enforced. On this occasion the highest solemnities of the law were observed. George II. issued a special commission to his Chancellor, Henly, reciting that the King considering justice an excellent virtue, and pleasing to the Most High, and concluded with making him Lord High Steward, with authority to preside in the august court thus organized. Upon the trial, the Solicitor-General, quoting the law as laid down by Hale (whom he terms the wise judge and great lawyer), says, that the result of his whole reasoning stands thus:—"If there be a total, permanent want of reason, it will acquit the prisoner. If there be a total temporary want of it when the offence was committed, it will acquit the prisoner; but if there be only a partial degree of insanity, mixed with a partial degree of reason; not a full and complete use of reason, but a competent use of it, sufficient to have restrained those passions which produced the crime; if there be thought and design; a faculty to distinguish the nature of actions, to discern the difference between moral good and evil; then, upon the fact of the offence proved, the judgment of the law must take place."

The case of James Hadfield, quite as interesting as the one first-mentioned, was tried in 1800. The indictment was for shooting at the king, &c., in a crowded theatre, just as he entered the box, and the audience was rising to cheer him. The rule as to responsibility for crime, was substantially the same as quoted above: though Mr. Erskine commented upon the rule insisted on by the Attorney-General, that to protect a man from criminal responsibility, there must be a total deprivation of memory and understanding. He admits it the very language of Coke and Hale, but contends it cannot be applied in a literal sense, for in that case such a thing as insanity seldom if ever occurred.

It appeared on the trial that the prisoner had been a soldier, and wounded in battle by a blow upon the head, breaking the skull and injuring the brain; that immediately after the wound was received, he became crazy, and continued so occasionally up to the time of his attempt to kill the king: his insanity being intermittent. Prior to his receiving the wound, the witnesses proved him brave and loyal, and the jury acquitted him on the ground of insanity.

It has been sometimes said that the law does not understand, or knows no distinction between different kinds of insanity. This is not strictly true, as is proved by the case of John Bellingham, tried for the murder of the Right Honourable Spencer Percival, before Chief Justice Mansfield, in 1812. The rule, as laid down in that case, exempts the prisoner from responsibility, provided he is found deprived of all power of reasoning, so as not to be able to distinguish whether it was right or wrong to commit the most wicked transaction. But this, he adds, must be proved; and the jury must find it as a fact, beyond all doubt, that at the time he committed the act with which he stood charged, he did not consider murder was a crime against the laws of God and nature. There was no other proof of insanity which would excuse murder or any other crime.

After speaking of other kinds of insanity, the judge then goes on to say:—"There was a third species of insanity, in which the patient fancied the existence of injury, and sought an opportunity of gratifying revenge by some hostile act. If such a person were capable, in other respects, of distinguishing right from wrong, there was no excuse for any act of atrocity, which he might commit under this description of derangement."

On the trial of Hadfield, mentioned above, it was contended by Mr. Erskine, on behalf of the prisoner, and may be assumed as admitted by the court, that where the prisoner laboured under a delusion connected directly with the subject-matter of the transaction for which he stands indicted, he cannot be convicted of crime, even though he be not deprived of all power of reasoning. This distinction, however, when examined, fades away into the original colour, and leaves to the jury still the same simple inquiry, whether the party charged with the offence *knew* that the very act he committed was criminal.

Having referred to a few of the leading cases on the subject of insanity, enough to show what the law now is, and how far it enforces human responsibility, we arrive at the point where we have a right, and are bound to speak for ourselves. With a proper estimate of history, we cannot be indifferent to the past, and those various influences out of which have arisen our present social relations. We go back to the sources of civilization with pleasure, and trace with delight the increasing and expanding volume as it emerges from the wild and mountainous regions of romance, and opens on the unobstructed plains of history. We listen to its many voices, and make ourselves acquainted with its wisdom. We go out of ourselves and the present time, to learn thoughts of those who have preceded us. We gather instruction from their deeds, and a wise forecast from their folly. It is thus we trace the progress of opinions, and the slow, though constant and firm, advance in the tone and temper of law—that high and sublime march of the people, in which there are few hasty changes, and no magnificent studies, but a modest and steady progression, keeping time with the music of intelligent thought. It is not a romance, nor an epic poem; it is no picture of the imagination, nor republic of Utopia; but a system of principles that sprung up out of the national mind, and adapted themselves to every condition and circumstance of life. Flexible in their nature, and always closely surrounding us, we are generally unmindful of their presence till the very moment we need protection, so easily and naturally do we wear them as an armour of defence.

Like our political institutions, they come down to us from the past, associated with the events and scenes of history: imperfect in particulars, but in the main breathing the earnest and manly spirit of times when men stood upon their rights, maintained the claims of the citizen against the sovereign, and established the law upon the rough and rugged field of battle. They come to us dressed in the style of an early day, but with a universal and catholic authority, comprehending the past, present, and future. They command respect and elicit our regard in infancy and childhood, long before we are able to understand them or appreciate their excellence. It is thus the common law becomes a part of the common mind, intimately blending itself with the thoughts, and entering into the judgments of each individual: so that it is not, perhaps, too much to say, that on general subjects the common opinion of the law is the highest and best evidence of what that law is.

There is a strange and wonderful interest attaching itself to every description of insanity. The subtile relation existing between the material and immaterial man, that intimate association of mind with body, acting and reacting sympathetically upon each other, is at all times a subject of interesting and curious speculation. But when examined in connexion with derangement of the mental powers, it becomes a mystery passing the ken of human knowledge, around which the light of science sheds no illumination, and gives token of no discovery. On other subjects, investigation repays us with a fixed and satisfactory result; we congratulate ourselves with the discovery of truth, and the establishment of those general principles upon which the sciences are based. It is a pleasure that springs out of certainty and system, and a harmony that rises from many voices mingling in unison.

But on this subject we have no system: it is all mysterious and uncertain, complex and wonderful, as are the operations of the human mind. For though we are able to understand many of the influences that operate remotely to induce insanity;—though we can speak of the phenomena that attend it, and sometimes point out the *causes* that seem to have produced it;—though we can trace its stages through disappointment, melancholy, wakefulness, and a sad brooding over real or imaginary wrong, observe the freaks of fancy, the odd conceits and strange devices that occasionally denote the source of madness;—though we can sometimes discover and pronounce upon the subject around which the brittle thread of reason was broken;—our skill is at fault, and fails us when we attempt to classify the causes, or speak with accuracy of a general origin of mental disease. Each case is so peculiar, it furnishes a law for itself.

In the tragedy of *Hamlet* it has long been a question among critics whether the great master intends to portray actual or assumed madness. Soon after seeing his father's ghost, we find him swearing his friend, Horatio, to silence and secrecy, intimating his intention "to put an antic disposition on," the better to cover his proceedings. Directly we hear him lamenting his feebleness and want of spirit in such a style as convinces us of the deep melancholy that has settled on his mind, and darkened his prospects. He is called to a mighty work, and feels himself incompetent to the task. His nature is noble; he has been accustomed to believe in the sincerity of his companions, and to trust the integrity of the king. He has been surrounded from infancy with flatterers, and those who have courted him as the heir-apparent to the throne. He has yielded himself to the protestations of friendship, and to the soft, winning accents of tenderness and love. The gaieties of life have thrown a charm around him, and his youth has passed away like the sweet influences of spring, the bloom and beauty of the year. He has not known disappointment, nor anticipated danger; the smooth current of his being has flowed like a river.

From such a life he is suddenly aroused to new thoughts. The death of his father was not natural—there was a strangeness about the circumstances, a solemn show of grief, a haste to close over the grave, and a grasping of the crown, that threw a shadow and a doubt over him that wore it. There are no witnesses to the deed—the act was done in silence. No eye saw it, and no tongue has spoken of it. But it was a bloody deed, and cries for vengeance. The ghost of the murdered man cannot rest in his grave, but wakes to walk the earth at night, and whisper of the foul treason; how he was cut off in the blossom of his sin, and sent to his account with all his imperfections on his head. "Unhousel'd, disappointed, unaneal'd." The manner of the murder is known, and Hamlet is commissioned to avenge the most foul and unnatural crime. Henceforth he is a new man; the pleasures of life pall on his taste, and the objects that have occupied his attention have been changed, as by the touch of magic, into the veriest baubles. His deep spirit has been stirred within him, and one great passion controls and masters every thought. His mind is unnaturally active, but his purposes are weak, and dispose him to meditation. He believes, and yet he doubts, and so devises a scheme to catch the conscience of the king, and assure himself that he is not beguiled by the devil; for he is still uncertain about the character of the fearful and dread apparition. In this state of suspense, everything becomes suspicious and questionable. The world is not what it used to be. Hamlet contemplates suicide, and runs over in his mind the prospects of a future life, the sleep of death, the dread of something after death, the clouds and darkness that hang over the undiscovered future: he then glances at the evils of the present life, and multiplies them, and magnifies

"The scorns of time,
The oppressor's wrong, the proud man's contumely,
The pangs of despised love, the law's delay,
The insolence of office, and the spurns
That patient merit of the unworthy takes."

By and by, in his interview with his mother, he undertakes to speak to her of her crimes, grows warm with the theme, utters words of burning sarcasm, bitter hatred, terrible and scathing rebuke. When in the very height of his passion and fiery denunciation, his father's ghost again appears, charging him

"Do not forget—this visitation
Is but to whet thy almost blunted purpose."

The mother observes his manner as he listens to the strange visitor, and questions him, that he bends his eye on vacancy, and holds discourse with the incorporeal air, and calls his vision the very coinage of his brain, an ecstasy. To this he indignantly replies—

"Ecstasy!
My pulse, as yours, doth temperately keep time,
And makes as healthful music; it is not madness
That I have utter'd; bring me to the test,
And I the matter will re-word, which madness
Would gambol from. Mother, for love of grace,
Lay not that flattering unction to your soul,
That not your trespass, but my madness speaks."

He is by turns desponding and energetic. When alone, he seems to question the source of his information, and wonders whether he is not acting under the instigation of some dark and mysterious agency. When in the presence of his mother or the king, no doubt any longer lingers about his mind. The enormity of the crime alone impresses him; his speech becomes impassioned, and he grows impatient of delay; but his stormy zeal seems to vent itself in vigorous and violent language, and resolution dies the moment he is left alone. In speech, like all madmen of his mind and temperament, he is perfectly terrible, but in action as weak and unsteady as a child. There is method in his madness, and he appears to act with a preconceived design; but for all that there is a fickleness and irresolution about him, and a wildness that casts suspicion over his whole character, and leaves us at times in doubt whether we are listening to the insane ravings of a madman possessed of a strange and mysterious plot, or following the course of an injured prince who seeks redress of a wrong beyond the power of the law, and justice upon the head that wears the crown.

We had intended to inquire somewhat carefully into the nature of insanity, the condition of mind, and real ability of the insane. But our limits on this occasion forbid us to do more than simply refer to the subject; and point out the fact that, among the insane there are but few, not more, perhaps, than one in a hundred, who are *totally* insane, so that a jury might with propriety pronounce them incapable of distinguishing between right and wrong. Most of those confined in our asylums are what we commonly call monomaniacs—their insanity being connected with particular subjects. They are insane on religious questions, on money matters, love affairs, and schemes of speculation: from sickness, disease of the brain, loss of friends, and a thousand other causes, some of which we are acquainted with, while others escape observation.

At present we confine our attention to the legal and moral responsibilities of the insane. And here, if we mistake not, had no rule ever been adopted, and the question were now for the first time presented, whether the law should make any distinction in its treatment of the insane, between what is termed partial and total insanity, there would, we apprehend, be but one opinion. The impossibility of drawing the line between them would alone be sufficient to demonstrate its impolicy, if not injustice. Besides, on a matter of so much moment and practical importance, a rule that is to be enforced ought to be clearly drawn, so that the distinction need not be left to the jury to make, according as their prejudices or the circumstances of the case may incline. The language of the law should be clear and definite, such as may not be misunderstood by judge or jury. As the rule now stands, the administration of it is exceedingly difficult; it is plain enough *theoretically*, but practically, infinitely difficult to be applied. The witness shows the conduct of the prisoner to be insane; the judge declares that if he be so insane as not to know what is right, he cannot be convicted of crime. Here the jury take the case with almost legislative powers, and set themselves to inquire about the prisoner's capacity to distinguish between good and evil, an inquiry, where insanity is shown, involving difficulties to the jury and dangers to the citizens, to which neither should be subjected under wise and just laws.

Now, under the old principle, as laid down by the early writers, it is quite possible that the law be rigidly enforced while the most monstrous injustice is perpetrated; and this fact alone demonstrates the propriety of such an amendment as will for ever render it impossible to commit so grievous a wrong in the sacred name of justice. Under the present decisions of our courts, they are understood to hold that an individual may be insane in respect to money affairs, and still capable of committing the crime of murder or arson, and so of all monomaniacs. On the immediate subject of their delusion, they are not considered moral agents; on all others they are held to a strict accountability. The man we saw at the asylum at Utica, who considered himself the great financial agent of the state, controlling the operations of Wall Street, and the slightest transactions in the market, coining gold and silver, and sending them forth as a convenient currency for the accommodation of the community—that man, under the legal rule, would not, perhaps, be deemed capable of theft or robbery. The particular nature of his delusion would render it impossible. Not so in reference to other subjects. True, it is thought by some that such an unsoundness destroys the idea of moral responsibility. The law, however, is more rigid and stoical; it holds there may be insanity and a moral sense still remaining in the mind with a responsible judgment, and makes the circumstances of each particular case determine whether the moral sense be entirely destroyed, or only affected by the moral unsoundness. If the individual labour under a single delusion that will not yield to evidence, and remain otherwise sane, the philosophy of the law, as at present expounded, assumes that, upon questions in which the delusive ideas are not necessarily involved, they will have no influence upon the mind; so that if there remain the bare knowledge of right and wrong, the person is capable of committing crime, no matter how strange and absurd may be the action of his passions.

The man Mr. Erskine mentioned in the Hadfield trial, who believed himself the Christ, evidently could distinguish right and wrong. His standing a severe cross-examination so long, baffling the utmost skill of counsel, as well as his complaints against the committee of his estate, showed his sense of justice, and that he appreciated, to some extent, his own rights and relations to others. But for all that, who would think of holding him capable of crime? He really believes himself the Saviour of mankind, and as such, empowered to forgive sins. Shall such a man be punished for the dreamy speculations and uncertain action of a shattered intellect? It would be a monstrous doctrine to maintain, and still more monstrous to enforce. And yet, under the rule, the jury must either make the law what the justice of the case requires, and thereby liberally construe the oath they take, to render a verdict according to the evidence, into a general obligation to do what is right in the particular case; or, they must find the unfortunate man guilty of a crime at which nature shudders.

The true rule, it should seem, would hold, that if a man be insane, the law ought to regard him as an infant, incapable of crime. It should not be a question whether he knows right from wrong, but whether he be sane or not; for if he be a monomaniac, he should not be punished, even though a jury be able to say, upon their oath, that he knew the act he performed was wrong. The association of ideas in the mind of the insane is too subtle for our comprehension, and the mystery of his motive too profound for our investigation. We assume to punish guilt, because we understand what constitutes crime in the case of a sane man; possessing, as we do, his thoughts and feelings, with enough of his motives to enable us to pronounce upon his conduct. But in respect to the insane, who knows the operations of his mind, or what dark power reigns over him? Who can enter into his spirit, or explore the labyrinth of his inconceivable thoughts? What can become so like him as to take upon himself *the very feelings of insanity*, and understand him as we understand each other? We are none of us able to do so. Would it not then be modest in us to waive a principle of law implying such knowledge?

In children, we frequently discover (or think we do) a knowledge of right and wrong, long before any man of sane judgment would think of holding them responsible for crime. The moral sense seems to grow with the faculties. It is at first feeble, its existence barely appearing to our observation. Gradually it becomes stronger, as the mind itself approaches the stature of manhood, so that the time when it assumes the guidance of conduct, and the child becomes capable of contracting guilt, is always doubtful and difficult to fix, depending, as it does, so directly upon the mental growth, the complete and harmonious development of each attribute and quality of mind. The moral sense—what is it indeed in any case but the simple judgment of a mind in which the intellect and sentiments unite in healthy activity? As we speak of it sometimes, a stranger to the common phraseology would think us talking of some imaginary being above and beyond us, when, in reality, we mean to discuss simply the mind's capacity of feeling and acting rightly; a capacity depending equally upon the natural action of the passions, and the perfect use of reason. This is our reasoning when we speak of children; why should we not apply the same principles, and allow ourselves to be governed by an equal sense of justice, when we come into the presence of reason-bereft and strangely-afflicted children of misfortune?—*American Review*, vol. viii. pp. 269—275. New York.

